# Determining the survival benefit of postoperative radiotherapy in patients with pT1-3N1M0 rectal cancer undergoing total mesorectal excision: a retrospective analysis

**DOI:** 10.1186/s12876-023-02697-4

**Published:** 2023-03-23

**Authors:** Jin-hu Chen, Qing Ye, Feng Huang

**Affiliations:** Department of Gastrointestinal Surgical Oncology, Fujian Cancer Hospital and Clinical Oncology School of Fujian Medical University, No. 420, Fuma Road, Jin’an District, Fuzhou, 350014 China

**Keywords:** Locally advanced rectal cancer, Adjuvant therapy, Postoperative radiotherapy, Total mesorectal excision

## Abstract

**Background:**

The National Comprehensive Cancer Network guidelines recommend routine postoperative adjuvant radiotherapy and chemotherapy for patients with stage III rectal cancer who do not receive neoadjuvant therapy before surgery. The present study aimed to evaluate the value of postoperative radiotherapy in patients with low-risk disease (pT1-3N1M0) who did not receive neoadjuvant therapy prior to total mesorectal excision.

**Methods:**

We used the Surveillance, Epidemiology, and End Results database (2004–2016) to retrospectively recruit patients with pT1-3N1M0 rectal cancer whose initial treatment was radical surgery with postoperative adjuvant chemotherapy. A propensity score model was used to balance the baseline covariates.

**Results:**

Of the 2012 patients included in the present study, 1384 received adjuvant chemoradiotherapy (radio group), whereas the remaining 718 received chemotherapy alone (no-radio group). There was no significant difference in cancer-specific survival rate between the two groups (log-rank test χ^2^ = 2.372, *P* = 0.124) in the overall sample. Additionally, in the propensity score−matched cohort, adjuvant radiotherapy did not improve cancer-specific survival. Subgroup analysis showed that having three positive lymph nodes and a tumor > 50 mm, combined with postoperative adjuvant chemotherapy, could lead to an improved tumor-specific survival rate, while other cases did not benefit from postoperative radiotherapy.

**Conclusions:**

For patients with pT1-3N1M0 rectal cancer who did not receive neoadjuvant therapy before surgery, postoperative radiotherapy in addition to adjuvant chemotherapy did not significantly improve survival rates. The number of positive nodes (*n* = 3) and tumor size (> 50 mm) were found to be potential screening indicators for postoperative adjuvant radiotherapy.

**Supplementary Information:**

The online version contains supplementary material available at 10.1186/s12876-023-02697-4.

## Background

Traditionally, postoperative adjuvant chemoradiotherapy has been administered to patients with locally advanced rectal cancer (LARC) who do not receive preoperative neoadjuvant therapy. Currently, however, the National Comprehensive Cancer Network (NCCN) recommends postoperative adjuvant radiotherapy and chemotherapy for almost all patients whose perioperative pathology results have confirmed their cancer to be pT3-4 or N1-2, except for some patients with pT3N0M0 cancers who have positive pathological prognostic features [1]. The aforementioned NCCN recommendations are primarily based on data from previous studies on postoperative adjuvant radiotherapy and chemotherapy for the treatment of rectal cancer, including NSABPR-01 [2], GTSG71-75 [3], and NCCTG79-47-51 [4]. However, these studies were completed in the 1990s. Currently, when using mesorectal integrity as the standard of evaluation, high-quality total mesorectal excision (TME) surgery results in a local recurrence rate of rectal cancer as low as 5% [5, 6]. However, it is unknown whether postoperative radiotherapy should be routinely implemented. In the present study, we evaluated the benefits of postoperative radiotherapy in addition to adjuvant chemotherapy after primary surgery in patients with pT1-3N1M0 rectal cancer who did not receive preoperative neoadjuvant therapy.

## Methods

### Data sources

This real-world study aimed to retrospectively analyze data from the Surveillance, Epidemiology, and End Results (SEER) program database. We used SEER Stat software (SEER*Stat 8.3.8, Incidence-SEER 18 Regs Custom Data with additional treatment fields, Nov 2018 Sub (1973–2016 varying)) to retrieve the data for all patients diagnosed with rectal cancer (C20.9 Rectum, NOS) between 2004 and 2016. A total of 8,324 patients with pT1-3M0 rectal cancer and regional nodes positive between 1 and 3 were included in the present analysis based on the American Joint Committee on Cancer staging system. Patients with multiple primary cancers (*n* = 1906), surgery type indicated as “no surgery performed,” “local tumor excision,” “proctectomy or proctocolectomy with an *en bloc* resection of other organs,” or “unclear” (*n* = 195), and/or radiation sequence with surgery recorded as “intraoperative rad with other rad before/after surgery,” “sequence unknown, but both were given,” “surgery both before and after radiation,” “radiation prior to surgery,” or “radiation before and after surgery” (*n* = 3,025) were excluded. Patients who did not receive adjuvant chemotherapy were also excluded (*n* = 1083), as were patients with survival times listed as “0” (*n* = 4) and patients with SEER cause-specific death classifications of “missing/unknown cause of death (COD)” (*n* = 9). Data from 2102 patients were included in the present study.

### Outcome and statistical analysis

The primary outcome variable in this study was cancer-specific survival. Categorical data were summarized using contingency tables, and the demographic and tumor characteristics of the patients were assessed using the chi-square or Fisher’s exact test. Cancer-specific survival rates were calculated, and the impact of radiation therapy was evaluated using Kaplan–Meier analysis. The log-rank test was used to determine whether the Kaplan–Meier survival curves were statistically equivalent. Additionally, we performed multivariate Cox proportional hazards regressions using sex, age, race, histological grade, tumor size, surgery type, T stage, number of resected nodes, number of positive nodes, and radiation as covariates. To make the baseline data of each subgroup comparable, nearest neighbor and 1-to-1 matching algorithms were performed within the default caliper (0.1) in SPSS version 25 (IBM, Chicago, IL). The matching variables included sex, age at diagnosis, race, tumor size, histological grade, surgery type, T stage, and N stage. We also cut points for categorical variables in the sub-analysis, including T stage, N stage, histological grade, and tumor size, to identify the effects of postoperative radiation. Lastly, hazard ratios (HR) were calculated using multivariate Cox proportional hazards regressions to assess the importance of postoperative radiation, and 5-year survival rates for different subgroups were calculated using Kaplan–Meier analysis. Statistical significance was set at *P* < 0.05, and statistical analyses were performed using the statistical software package SPSS for Windows, version 25 (IBM SPSS Inc., Chicago, IL).

## Results

### Patient characteristics

We retrospectively analyzed data of 2,102 patients with pT1-3N1M0 rectal cancer from the SEER database who did not undergo preoperative neoadjuvant therapy. All patients had undergone radical surgery (R0) and adjuvant chemotherapy; 1384 received adjuvant chemoradiotherapy (radio group), and the remaining 718 received chemotherapy alone (no-radio group). Systematic differences were noted in the overall samples, particularly in the demographic and clinicopathological characteristics, between the radio and non-radio groups. Compared to patients who did not undergo radiotherapy, those who more frequently underwent abdominoperineal resection (APR) had fewer lymph nodes retrieved and were typically diagnosed earlier (*P* < 0.001 for all; Table 1). The baseline characteristics in the overall sample were unbalanced between the two groups, and in order to obtain a balanced distribution of these baseline covariates, a propensity score model (1:1 ratio) was utilized. After propensity score matching, there were no significant differences in the pairwise comparisons of all covariates, indicating that the characteristics between the two treatment groups were balanced. This matched cohort included 842 patients, with an equal number of patients in the radio and no-radio groups (421:421).

**Table 1:** Demographic and clinicopathological characteristics of 2102 rectal cancer patients

Variables	No-radio (*n* = 718)	Radio (*n* = 1384)	*P* value
Gender			0.094
Male	386 (53.8%)	797 (57.6%)	
Female	332 (46.2%)	587 (42.4%)	
Age, Y			0.757
≤ 60	384 (53.5%)	750 (54.2%)	
> 60	334 (46.5%)	634 (45.8%)	
Year of diagnosis			0.000
2004–2009	303 (42.2%)	794 (57.4%)	
2010–2016	415 (57.8%)	590 (42.6%)	
Race			
White	583 (81.2%)	1117 (80.7%)	0.451
Black	56 (7.8%)	121 (8.7%)	
Other	79 (11.0%)	146 (10.5%)	
Grade			0.200
Grade I/II	592 (82.5%)	1125 (81.3%)	
Grade III/IV	112 (15.6%)	213 (15.4%)	
Unknown	14 (1.9%)	46 (3.3%)	
Histology			0.846
Adenocarcinoma	672 (93.6%)	1295 (93.6%)	
Mucinous & SRCC*	40 (5.6%)	74 (5.3%)	
Others	6 (0.8%)	15 (1.1%)	
Tumor size (mm)			0.929
≤ 50	510 (71.0%)	989 (71.5%)	
> 50	149 (20.8%)	278 (20.1%)	
Unknown	59 (8.2%)	117 (8.5%)	
Surgical type			
Sphincter preservation	640 (89.1%)	1154 (83.4%)	0.000
APR	78 (10.9%)	230 (16.6%)	
T stage			0.151
T1	127 (17.7%)	200 (14.5%)	
T2	175 (24.4%)	349 (25.2%)	
T3	416 (57.9%)	835 (60.3%)	
Number of nodes resected			0.000
< 12	147 (20.5%)	388 (28.0%)	
≥ 12	571 (79.5%)	996 (72.0%)	
Number of nodes positive			0.147
1	382 (53.2%)	675 (48.8%)	
2	210 (29.2%)	451 (32.6%)	
3	126 (17.5%)	258 (18.6%)	

*SRCC Signet ring cell carcinoma

### Cancer-specific survival analysis in overall samples

**Fig. 1:**
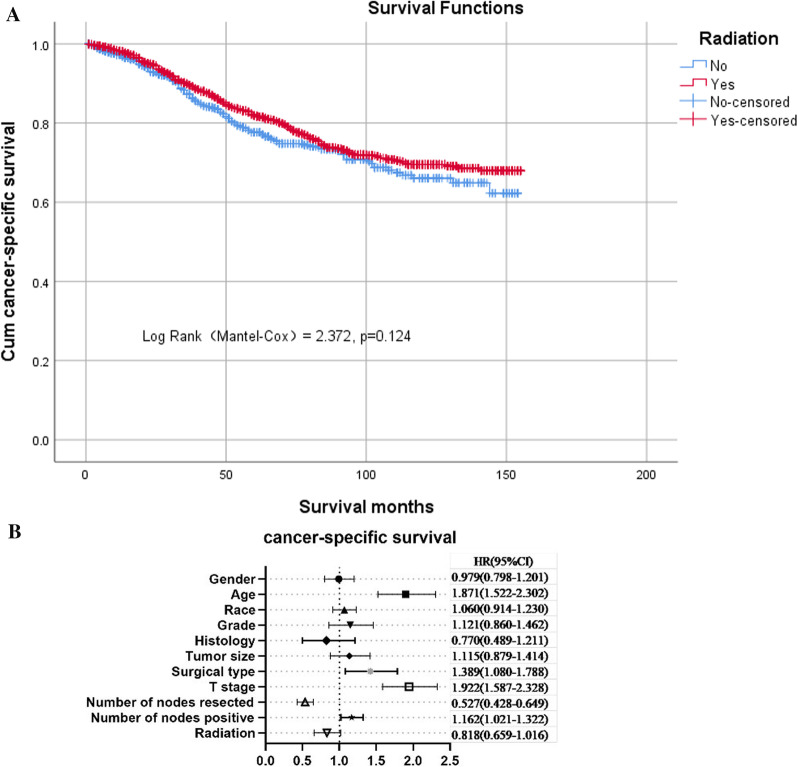
**A** Kaplan–Meier survival plots of cause-specific survival for the entire sample. **B** Hazards ratio (HR) of every clinical covariate from the Cox regression model for the entire sample

The median follow-up time for all patients was 60 months (range, 1–155 months). Of the 2102 patients included in our analysis, 438 died of rectal cancer; 145 (33.1%) were in the no-radio group, while the remaining 293 (66.9%) were in the radio group. Of the surviving patients and non-cancer-specific deaths, 941 had a follow-up period of > 60 months. Kaplan–Meier survival analysis showed that there was no significant difference in cancer-specific survival rates between patients who received adjuvant radiotherapy and those who did not (log-rank test χ^2^ = 2.372, *P* = 0.124, Fig. 1A). The 3- and 5-year cancer-specific survival rates were 89.9% and 81.9% in the radio group, and 87.3% and 77.6% in the no-radio group, respectively. Multivariate analysis also confirmed that adjuvant radiotherapy did not provide additional survival benefits in the overall cohort. Cox regression analysis also demonstrated that older age, APR, a higher T stage, and an increased number of positive lymph nodes all reduced survival rates, while an increased number of resected lymph nodes seemed to improve survival rates (Fig. 1B).

### Survival effects of postoperative radiotherapy in propensity score-balanced cohort

**Fig. 2:**
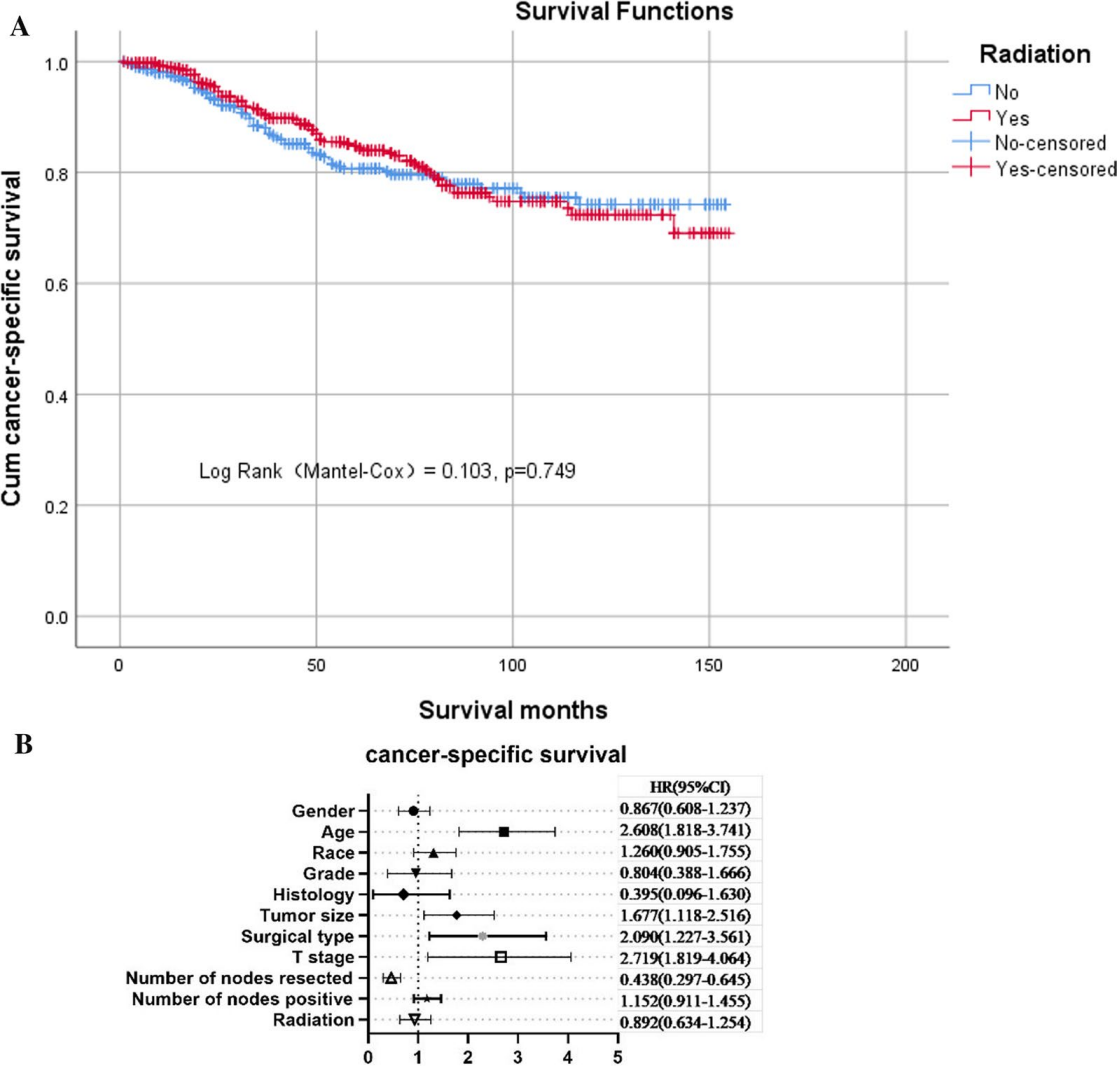
**A** Kaplan–Meier survival plots of cause-specific survival in the propensity score–matched cohort. **B** Hazards ratio (HR) of every clinical covariate from the Cox regression model in the propensity score–matched cohort

Of the 842 patients included in the propensity score–matched cohort, 139 died of rectal cancer, 68 (48.9%) were in the no-radio group and 71 (51.1%) were in the radio group. In the propensity score–matched cohort, adjuvant radiotherapy did not improve cancer-specific survival rates. The 5-year cumulative cancer-specific survival rates were estimated to be 84.3% and 80.7% in those who received postoperative radiotherapy and those who did not, respectively (log-rank test χ^2^ = 0.103, *P* = 0.749, Fig. 2A). Multivariate analysis also confirmed that postoperative radiotherapy did not provide an additional survival benefit in the propensity-score–matched cohort. Cox regression analysis demonstrated an HR of 0.892 for cancer-specific survival (95% confidence interval [CI] 0.634–1.254, Fig. 2B) for patients who received postoperative radiotherapy.

### Impact of postoperative radiotherapy on survival rates in different clinical subgroups from the overall sample

After overall analysis, we stratified the patients according to their T stage, number of positive lymph nodes, tumor size, grade, and histology, and identified the effects of postoperative radiotherapy on cancer-specific survival rates of different subgroups.

With regard to T stage, there was no significant difference in cancer-specific survival rates between the radio and no-radio groups for the T1, T2, and T3 subgroups (Additional file 1: Figure 3A, Additional file 2: Figure 3B, Additional file 3: Figure 3C). In T1-stage patients (*n* = 327), the 5-year cancer-specific survival rates were 93.3% and 90.2% in the radio- and no-radio groups, respectively. In T2-stage patients (*n* = 524), the 5-year cancer-specific survival rates were 88.9% and 86.0% in the radio and no-radio groups, respectively. In T3-stage patients (*n* = 1251), the 5-year cancer-specific survival rates were 76.3% and 70.6% in the radio and no-radio groups, respectively. The multivariate Cox regression model also did not show significant survival benefits for postsurgical radiotherapy in any of the T-stage subgroups (Additional file 4: Figure 3D).

**Fig. 3:**
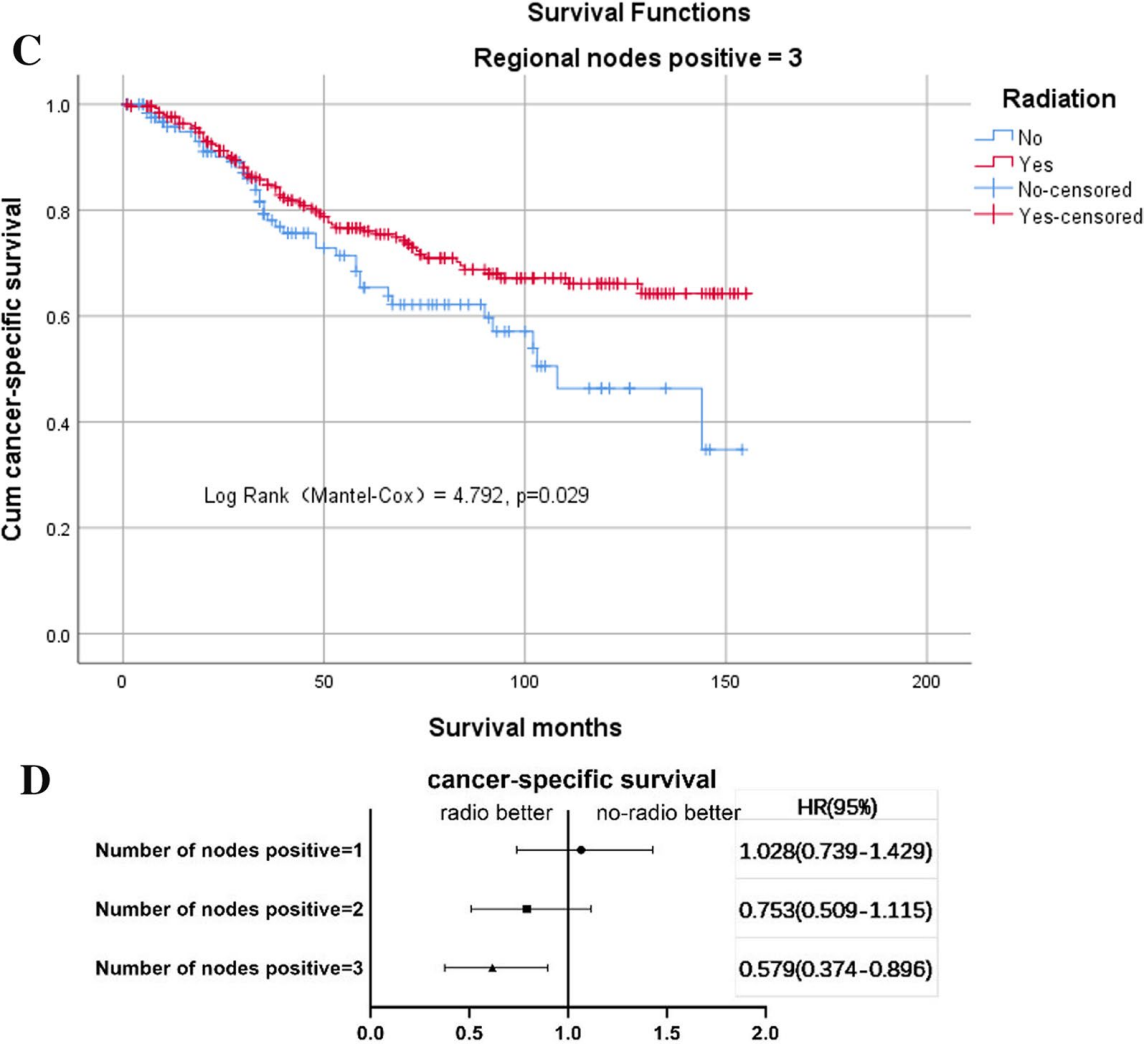
**C** Kaplan–Meier curves for cancer-specific survival (CSS) between radio and no-radio groups with three positive lymph nodes. **D** Risk ratio for different numbers of lymph nodes

In terms of N stage, a cancer-specific survival benefit from the addition of postoperative radiotherapy was only seen in the group with three positive lymph nodes (*n* = 384), with 5-year cancer-specific survival rates of 76.1% in the radio group and 65.4% in the no-radio group (log-rank test χ^2^ = 4.792, *P* = 0.029, Fig. 3C). In the group with one positive lymph node (*n* = 1057), the 5-year cancer-specific survival rates were 83.0% and 82.6% in the radio and no-radio groups, respectively (Additional file 5: Figure 4A), while in the group with two positive lymph nodes (*n* = 661), the 5-year cancer-specific survival rates were 83.7% and 75.9% in the radio and no-radio groups, respectively (Additional file 6: Figure 4B). The multivariate Cox regression model showed a significant survival benefit with the addition of postoperative radiotherapy only in the group with three positive lymph nodes (Fig. 3D).

**Fig. 4:**
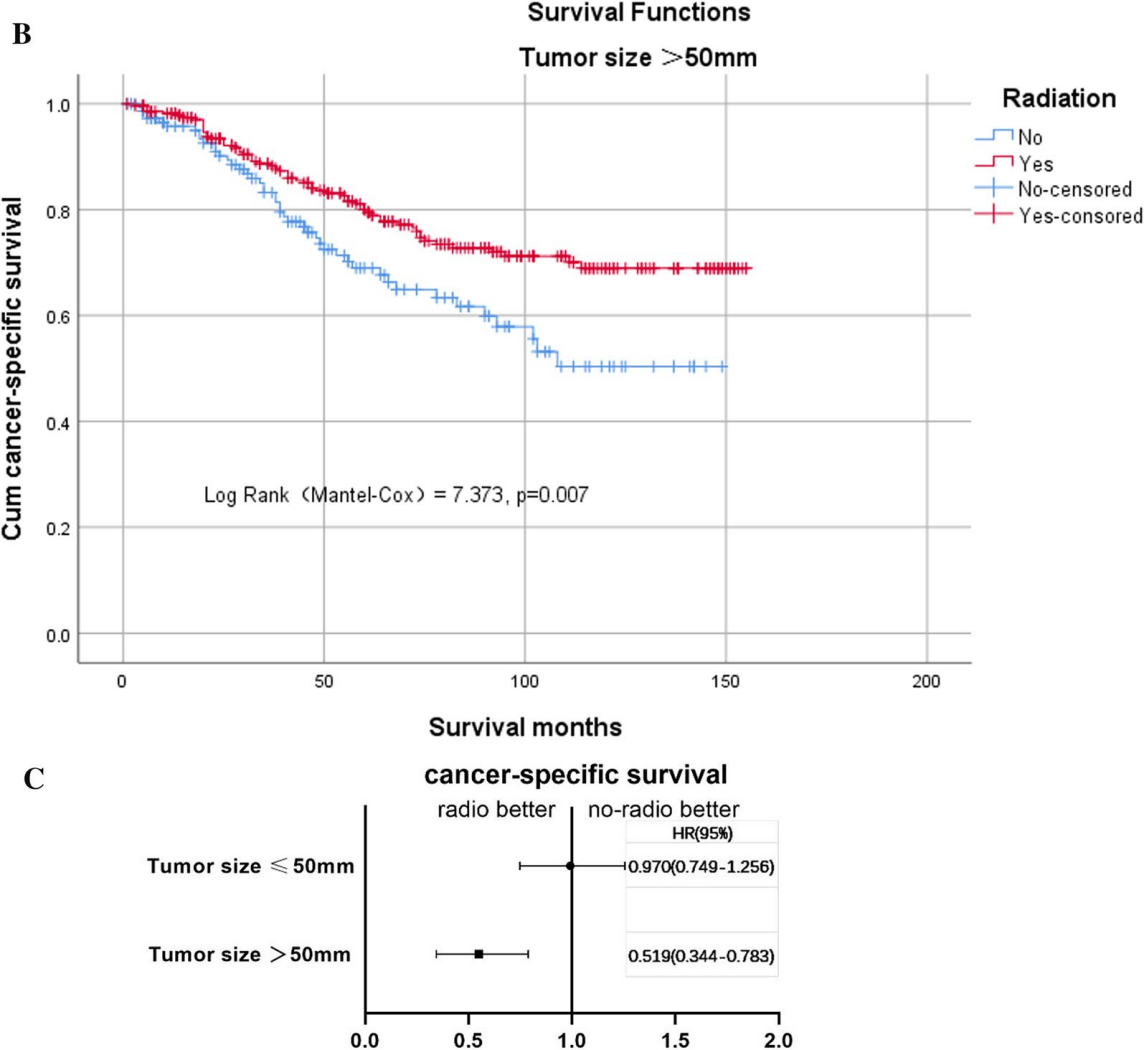
**B** Kaplan–Meier curves for cancer-specific survival (CSS) between radio and no-radio groups with a tumor size > 50 mm. **C** Risk ratio of different tumor sizes

In terms of tumor size, we did not observe a survival benefit for adjuvant postoperative radiotherapy when the tumor size was ≤ 50 mm (Additional file 7: Figure 5A). In contrast, in the group with tumors > 50 mm (*n* = 427), we found that postoperative radiotherapy improved the tumor-specific survival rate, with 5-year cancer-specific survival rates of 80.0% and 69.0% in the radio-and no-radio groups, respectively (log-rank test χ^2^ = 7.373, *P* = 0.007, Fig. 4B). We then adjusted for other covariates using the Cox regression model and obtained the same results: when the tumor size was > 50 mm, postoperative radiotherapy resulted in survival benefits (Fig. 4C).

In terms of histological grade, postoperative radiotherapy did not provide a survival benefit in either the grade I/II or grade III/IV subgroups (log-rank test χ^2^ = 1.186, *P* = 0.276; log-rank test χ^2^ = 0.672, *P* = 0.412, respectively; Additional file 8: Figure 6A, Additional file 9: Figure 6B). For the grade I/II group (*n* = 1717), the 5-year cancer-specific survival rates were 82.9% in the radio group and 78.7% in the no-radio group, whereas in the grade III/IV group (*n* = 1717), the 5-year cancer-specific survival rates were 75.4% and 72.3% in the radio and no-radio groups, respectively. The multivariate Cox regression model also did not show significant survival benefits for postoperative radiotherapy in the grade I/II and grade III/IV subgroups (Additional file 10: Figure 6C).

Postoperative radiotherapy did not play a significant role in terms of survival rate in either subgroup evaluated based on histology (Additional file 11: Figure 7A, Additional file 12: Figure 7B). In patients with adenocarcinoma (*n* = 1967), the 5-year cancer-specific survival rates were 82.1% in the radio group and 78.1% in the no-radio group (log-rank test χ^2^ = 1.979, *P* = 0.159), whereas in the mucinous adenocarcinoma and signet ring cell carcinoma (SRCC) group (*n* = 1717), the 5-year cancer-specific survival rates were 84.3% in the radio group and 77.7%, respectively (log-rank test χ^2^ = 0.212, *P* = 0.645). The multivariate Cox regression model also did not show significant survival benefits from postoperative radiotherapy in the adenocarcinoma, mucinous adenocarcinoma, and SRCC subgroups (Additional file 13: Figure 7C).

## Discussion

In 1982, Heald et al. [7] proposed that TME reduces the local recurrence rate of rectal cancer to approximately 10%. However, this idea was not accepted by most surgical experts around the world until the end of the twentieth century, when it became the gold standard for treatment of middle and lower rectal cancers. Subsequent studies, such as CAO/ARO/AIO-94 [8, 9], CKVO9504 [10], and MRCCR07 [11], showed that preoperative neoadjuvant radiotherapy and chemotherapy further reduced the local recurrence rate of LARC after radical resection to approximately 5%. Since then, preoperative neoadjuvant radiotherapy in conjunction with TME has become the standard treatment plan for middle- and lower-grade LARC. Although it reduces the local recurrence rate by approximately 5%, preoperative radiotherapy does not improve the overall survival (OS). In view of the fact that long-term adverse reactions caused by radiotherapy seriously affect patient quality of life by impairing anal, voiding, and sexual functions, injuries that can still increase with the passage of time, potentially increasing the risk of developing a second tumor [12, 13], some scholars have attempted to remove radiotherapy from the perioperative treatment of LARC.

The Chinese FOWARC study [14] was the first phase III clinical randomized controlled trial to introduce preoperative neoadjuvant chemotherapy into a neoadjuvant therapy plan for the treatment of rectal cancer. The results showed no significant differences in disease-free survival (DFS) and local recurrence rates between the perioperative mFOLFOX6 chemotherapy regimen without radiotherapy and standard fluorouracil-based neoadjuvant radiotherapy and chemotherapy. The 3-year local recurrence rates in the two groups were 8.3% and 8.0%, respectively. The aforementioned study speculated that mFOLFOX6 chemotherapy may be used as the initial regimen of neoadjuvant therapy for LARC, and radiotherapy can be used as a selective supplement based on the efficacy of chemotherapy. The American PROSPECT study [15] was the most anticipated study on neoadjuvant chemotherapy for rectal cancer, as it was a head-to-head control study that aimed to compare neoadjuvant therapy using a simple mFOLFOX6 regimen with standard neoadjuvant radiotherapy and chemotherapy. The results of the aforementioned study will have a decisive impact on the status of radiotherapy in the perioperative treatment of LARC and whether the comprehensive treatment of LARC will enter the era of neoadjuvant chemotherapy. The results of the FOWARC study posed a strong challenge to the perioperative use of radiotherapy for LARC.

One retrospective study found that the 10-year local control rate and recurrence-free survival rate of pT3N0M0 rectal cancer with good prognostic pathological features were 95% and 87%, respectively, whereas those of patients without good prognostic pathological features were 71% and 55%, respectively. This study suggests that postoperative adjuvant therapy has little benefit for pT3N0M0 rectal cancer with good prognosis [16]. Based on the aforementioned study, the NCCN guidelines recommend follow-up observation for patients with pT3N0M0 rectal cancer with good prognostic characteristics, whereas routine postoperative adjuvant radiotherapy and chemotherapy are recommended for patients with postoperative pathology confirmed as pT3-4 or N1-2 tumors.

The results of the present study show that in the era of relatively established TME procedures (2004–2016), radiotherapy based on the efficacy of adjuvant chemotherapy does not improve tumor-specific survival in rectal cancer patients with low-risk disease (pT1-3N1M0). To eliminate bias, we matched the baseline clinical data through propensity score balancing, after which we obtained the same results. In a real-world study, these results suggest that postoperative adjuvant radiotherapy does not significantly improve the prognosis of pT1-3N1M0 rectal cancer in conjunction with TME, which is an effective treatment for patients with pT1/pT2, pT3, and pN1 rectal cancer. If there is good quality smooth intact mesorectum post-TME, postoperative chemoradiotherapy is ineffective and unnecessary, as recommended by the European Society for Medical Oncology (ESMO) [17].

However, not all patients with pT1-3N1M0 rectal cancer can not benefit from postoperative radiotherapy. The subgroup analysis in the present study showed that having three positive lymph nodes and a tumor size > 50 mm, in combination with postoperative adjuvant chemotherapy, could result in improved tumor-specific survival rates, whereas other patients may not benefit from postoperative radiotherapy. Therefore, postoperative chemoradiotherapy (CRT) is only suitable for some LARC patients with poor selective prognoses, and the key lies in screening these patients. As an international pioneer in the individualized treatment of rectal cancer, the 2017 edition of the ESMO guidelines for rectal cancer points out that routine use of CRT is not necessary for LARC that has not been treated preoperatively if high-quality TME can be guaranteed. Postoperative CRT may be selectively used in patients with adverse histopathological features after primary surgery.

The ESMO guidelines specify four recommendations for postoperative adjuvant radiotherapy and chemotherapy: ① sufficient and necessary—circumferential resection margin (CRM) ≤ 1 mm, pT4b or pN2, extracapsular spread close to the mesorectal fascia (MRF), extranodal deposits (N1c), or pN2 if poor mesorectal quality/defects; ② sufficient—lower pN2 tumors within 4 cm of the anal verge (risk of involved lateral pelvic lymph node) or extensive extramural vascular or perineural invasion close to the MRF; ③ borderline sufficient—pN2 in the middle or upper rectum if good mesorectal tissue quality, CRM 1–2 mm, or circumferential obstructing tumors; and ④ insufficient and unnecessary—pT1/pT2, pT3, CRM > 2 mm, pT4a above reflection, or pN1 if good quality, smooth, intact mesorectum.

The ESMO guideline-based recommendations are largely based on the MERCURY study, in which patients with a good prognosis based on high-resolution magnetic resonance imaging (MRI) were treated solely with TME, with no adjuvant radiotherapy performed before or after the operation. A total of 374 patients were enrolled in the study, of which 122 (33%) had a 5-year local recurrence rate of 3%. Subgroup analysis showed that the local recurrence rate of stage T3 rectal cancer with good prognosis, as assessed by MRI (invasion of the mesorectum < 5 mm, regardless of N stage), was only 1.7% [5]. The results of the MERCURY study suggest that radiotherapy is likely to have no value in T3a or T3bN rectal cancers. Taylor et al. [6] reported the 5-year follow-up results of all cases included in the MERCURY study as follows: based on high-resolution MRI, all cases were divided into the MRI-clear CRM and MRI-involved CRM groups; predicted clear mrCRM was defined as the distance from the tumor to the MRF > 1 mm, while for lower-third rectal tumors, mrCRM involvement was defined as tumor ≤ 1 mm from the levator muscle. If the tumor was present at or below the level of the puborectalis sling, the mrCRM was predicted to be involved if there was invasion into the intersphincteric plane or beyond. A total of 205 patients in the MRI-clear CRM group underwent TME without postoperative adjuvant radiotherapy, and 58% of the patients were treated with adjuvant chemotherapy. Of these patients, the pathological circumferential resection margin (pCRM) was negative in 190 cases, and local recurrence occurred in 11 cases during the follow-up period. pCRM was positive in 15 cases, and local recurrence occurred in 3 of these cases during the follow-up period. The overall local recurrence rate was 6.8%, whereas the local recurrence rate for 190 cases with negative CRMs was only 5.8%. In the MERCURY study, the quality of TME was strictly controlled, and the aforementioned ESMO guideline-based recommendations strongly depend on the quality of the surgery, as a smooth and intact mesorectum is necessary to negate the need for radiotherapy. Therefore, the quality of the operation is an important factor when deciding whether to use radiotherapy. For patients with LARC who do not receive preoperative neoadjuvant therapy, if high-quality TME can be guaranteed, routine postoperative CRT should only be selectively utilized in patients with poor histopathological features after primary surgery. The ESMO guidelines indicate that adverse histopathological features include CRM ≤ 1 mm, pT4b, pN2, extracapsular spread close to the MRF, extranodal deposits (N1c), pN2 if poor mesorectal quality/defects, and extensive extramural vascular or perineural invasion close to the MRF. Based on the results of the present study, the presence of 3 positive lymph nodes and a tumor size > 50 mm should be regarded as poor histopathological features, indicative of sufficient conditions for the utilization of postoperative radiotherapy. The results of the present study are applicable to patients whose preoperative staging was underestimated and those who did not receive preoperative neoadjuvant radiotherapy or chemotherapy. Preoperative neoadjuvant radiotherapy and chemotherapy are the first choices for the treatment of newly diagnosed LARC, including total neoadjuvant treatment (TNT).

There are several limitations in this study. Some patients might not have undergone neoadjuvant treatment or postoperative adjuvant radiotherapy because of comorbidity, so this study has a selection bias. Since the present study was retrospective in nature, operation details and the quality of the postoperative rectal specimens are unknown. This study reflects real-world data, but is a study spanning 12 years, during which time surgical techniques and adjuvant chemotherapy regimens may have changed. Therefore, there may be a potential offset. In the post-TME era, multicenter phase III clinical studies on postoperative adjuvant radiotherapy for rectal cancer are still needed to confirm our findings.

## Conclusion

In conclusion, the results of the present study show that for the overall cohort of patients with pT1-3N1M0 rectal cancer, radiotherapy in addition to postoperative adjuvant chemotherapy did not significantly improve survival. The poor histopathological features proposed in the ESMO guidelines are important indicators for postoperative adjuvant radiotherapy screening; the number of positive nodes (3) and tumor size (> 50 mm) may also be screening indicators.

## Supplementary Information

**Additional file 1. Figure 3A.** Kaplan-Meier curves for cancer-specific survival (CSS) between radio and no-radio groups at T1.

**Additional file 2. Figure 3B.** Kaplan-Meier curves for cancer-specific survival (CSS) between radio and no-radio groups at T2

**Additional file 3. Figure 3C.** Kaplan-Meier curves for cancer-specific survival (CSS) between radio and no-radio groups at T3.

**Additional file 4. Figure 3D.** Risk ratio of different T stages

**Additional file 5. Figure 4A.** Kaplan-Meier curves for cancer-specific survival (CSS) between radio and no-radio groups with one positive lymph.

**Additional file 6. Figure 4B.** Kaplan-Meier curves for cancer-specific survival (CSS) between radio and no-radio groups with two positive lymph

**Additional file 7. Figure 5A.** Kaplan-Meier curves for cancer-specific survival (CSS) between radio and no-radio groups with a tumor size ≤ 50mm

**Additional file 8. Figure 6A.** Kaplan-Meier curves for cancer-specific survival (CSS) between radio and no-radio groups with grade I/II

**Additional file 9. Figure 6B.** Kaplan-Meier curves for cancer-specific survival (CSS) between radio and no-radio groups with grade III/IV

**Additional file 10. Figure 6C.** Risk ratio of different histological grade

**Additional file 11. Figure 7A.** Kaplan-Meier curves for cancer-specific survival (CSS) between radio and no-radio groups in adenocarcinoma.

**Additional file 12. Figure 7B.** Kaplan-Meier curves for cancer-specific survival (CSS) between radio and no-radio groups in mucinous & SRCC

**Additional file 13. Figure 7C.** Risk ratio of different histological type.

## Data Availability

The datasets generated and analyzed during the current study are available in the SEER database (https://seer.cancer.gov/) and from the corresponding authors upon reasonable request.

## Abbreviations

APR: Abdominoperineal resection
COD: Cause of death
CRM: Circumferential resection margin
CRT: Chemoradiotherapy
CSS: Cancer-specific survival
DFS: Disease-free survival
ESMO: European Society for Medical Oncology
HR: Hazard ratio
LARC: Locally advanced rectal cancer
MRF: Mesorectal fascia
MRI: Magnetic resonance imaging
mrCRM: MRI circumferential resection margin
NCCN: National Comprehensive Cancer Network
OS: Overall survival
pCRM: Pathological circumferential resection margin
SEER: Surveillance, Epidemiology, and End Results
SRCC: Signet ring cell carcinoma
TME: Total mesorectal excision
TNT: Total neoadjuvant treatment
