# Supplementary figures and images for: Determining the survival benefit of postoperative radiotherapy in patients with pT1-3N1M0 rectal cancer undergoing total mesorectal excision: a retrospective analysis
Source: BMC Gastroenterol. 2023 Mar 23;23:83. doi: 10.1186/s12876-023-02697-4 (PMC10037866; doi:10.1186/s12876-023-02697-4)

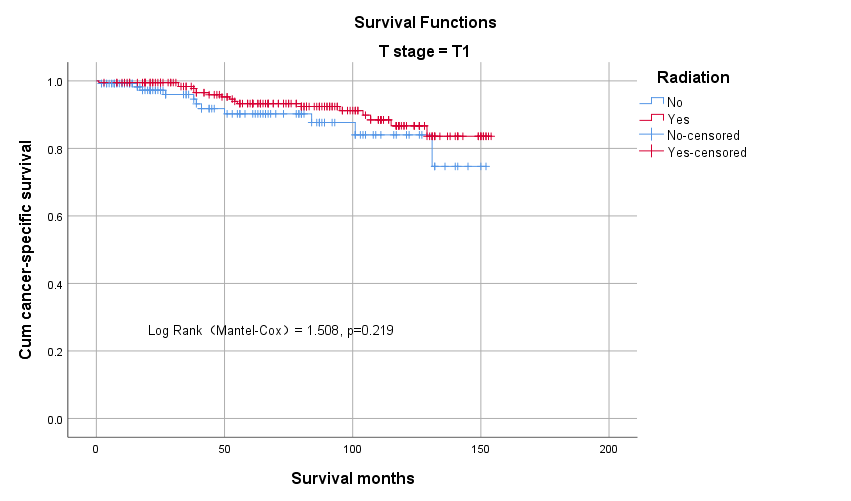

Supplement: Supplementary file 1 — Additional file 1. Figure 3A. Kaplan-Meier curves for cancer-specific survival (CSS) between radio and no-radio groups at T1. [file 12876_2023_2697_MOESM1_ESM.png]

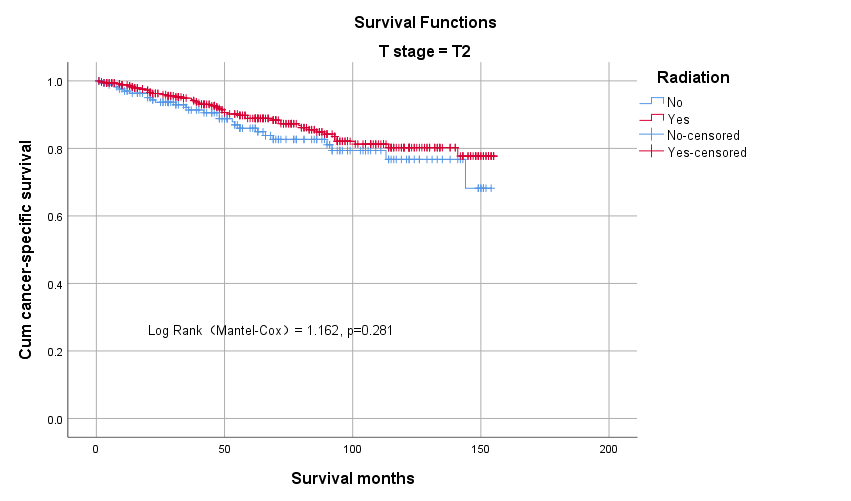

Supplement: Supplementary file 2 — Additional file 2. Figure 3B. Kaplan-Meier curves for cancer-specific survival (CSS) between radio and no-radio groups at T2 [file 12876_2023_2697_MOESM2_ESM.png]

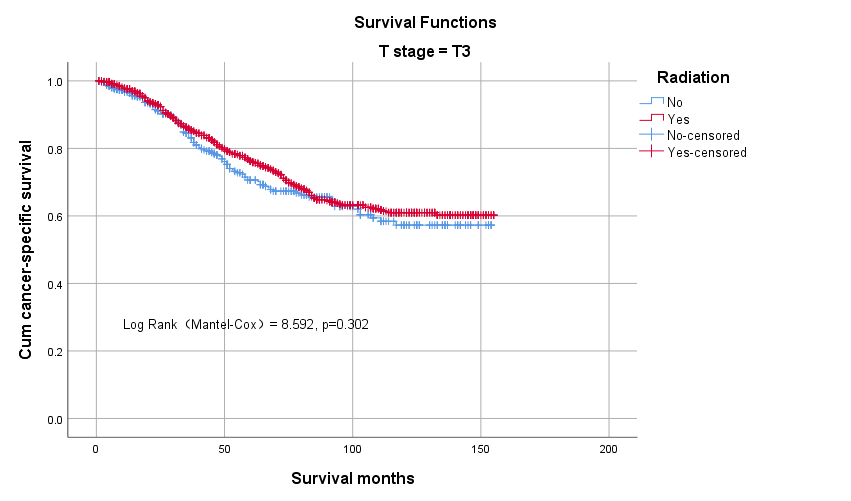

Supplement: Supplementary file 3 — Additional file 3. Figure 3C. Kaplan-Meier curves for cancer-specific survival (CSS) between radio and no-radio groups at T3. [file 12876_2023_2697_MOESM3_ESM.png]

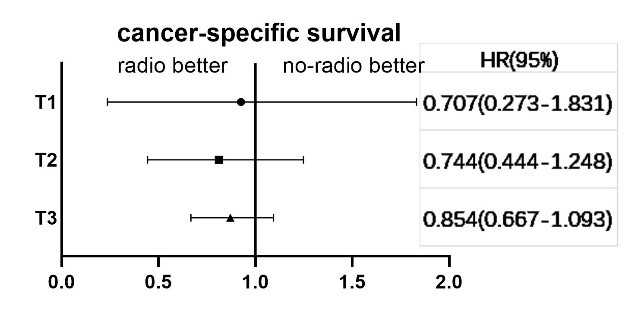

Supplement: Supplementary file 4 — Additional file 4. Figure 3D. Risk ratio of different T stages [file 12876_2023_2697_MOESM4_ESM.jpg]

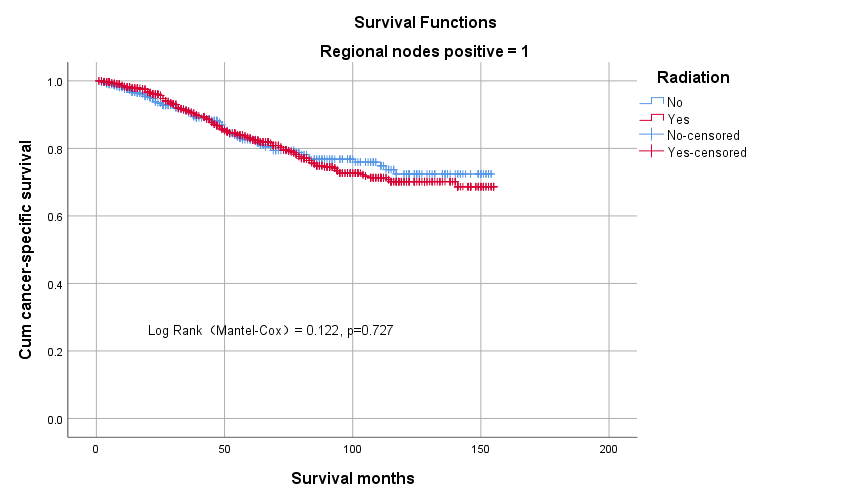

Supplement: Supplementary file 5 — Additional file 5. Figure 4A. Kaplan-Meier curves for cancer-specific survival (CSS) between radio and no-radio groups with one positive lymph. [file 12876_2023_2697_MOESM5_ESM.png]

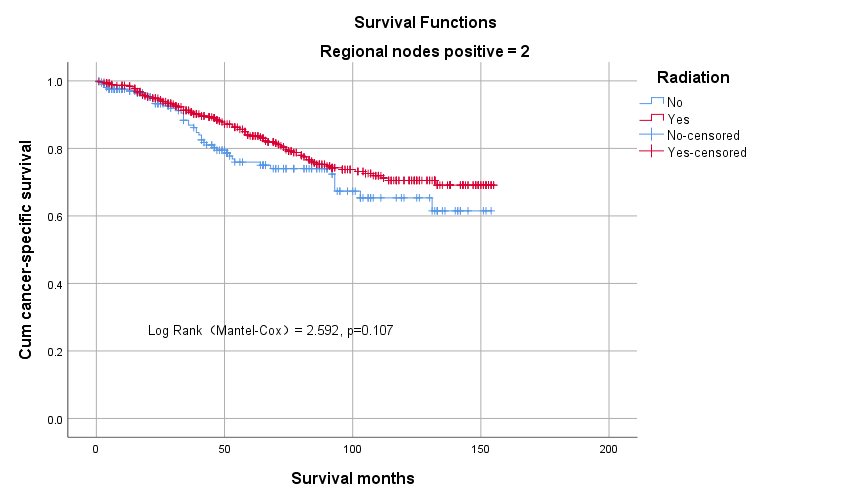

Supplement: Supplementary file 6 — Additional file 6. Figure 4B. Kaplan-Meier curves for cancer-specific survival (CSS) between radio and no-radio groups with two positive lymph [file 12876_2023_2697_MOESM6_ESM.png]

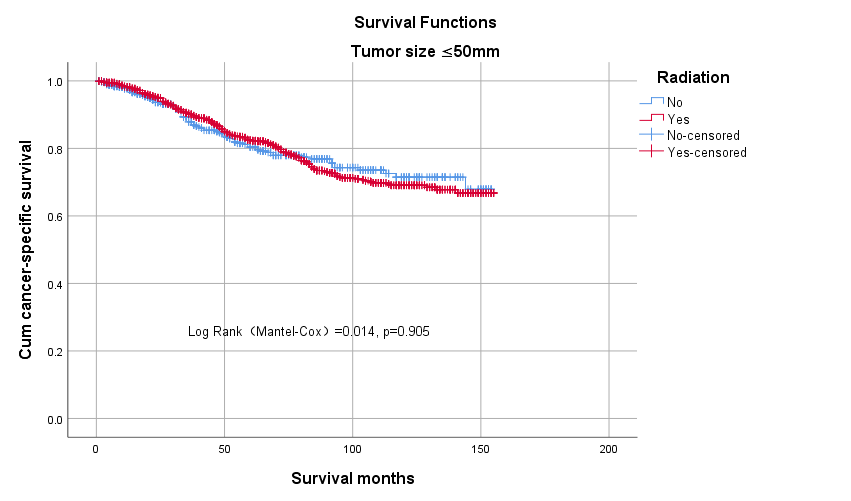

Supplement: Supplementary file 7 — Additional file 7. Figure 5A. Kaplan-Meier curves for cancer-specific survival (CSS) between radio and no-radio groups with a tumor size ≤ 50mm [file 12876_2023_2697_MOESM7_ESM.png]

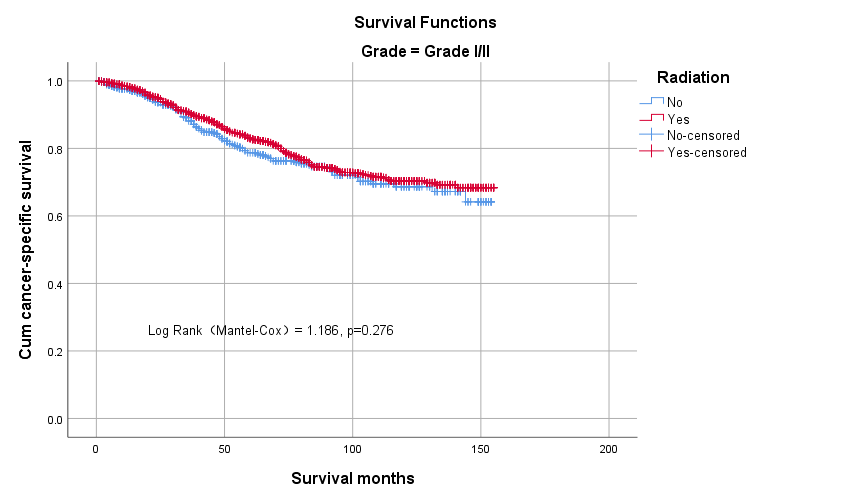

Supplement: Supplementary file 8 — Additional file 8. Figure 6A. Kaplan-Meier curves for cancer-specific survival (CSS) between radio and no-radio groups with grade I/II [file 12876_2023_2697_MOESM8_ESM.png]

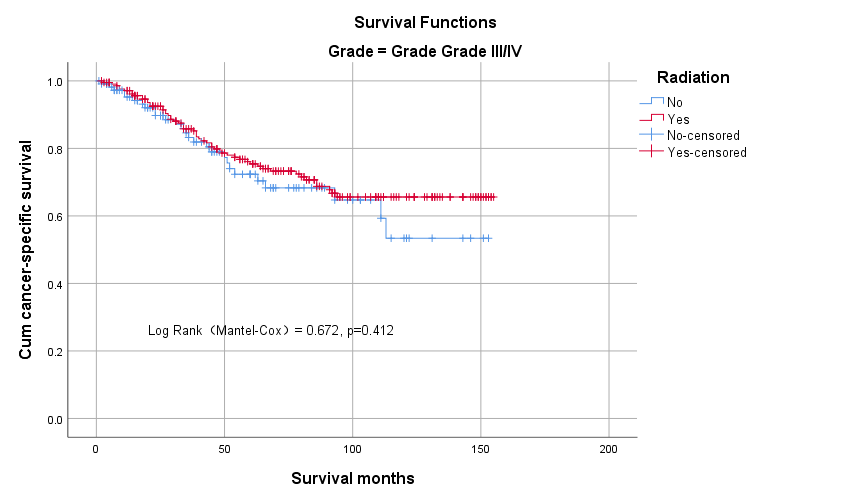

Supplement: Supplementary file 9 — Additional file 9. Figure 6B. Kaplan-Meier curves for cancer-specific survival (CSS) between radio and no-radio groups with grade III/IV [file 12876_2023_2697_MOESM9_ESM.png]

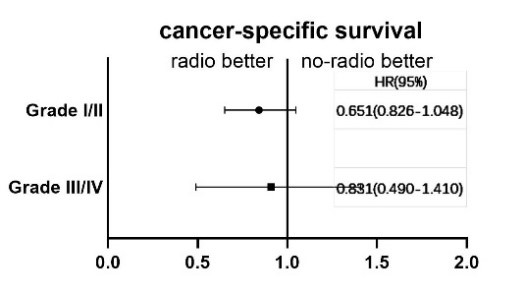

Supplement: Supplementary file 10 — Additional file 10. Figure 6C. Risk ratio of different histological grade [file 12876_2023_2697_MOESM10_ESM.jpg]

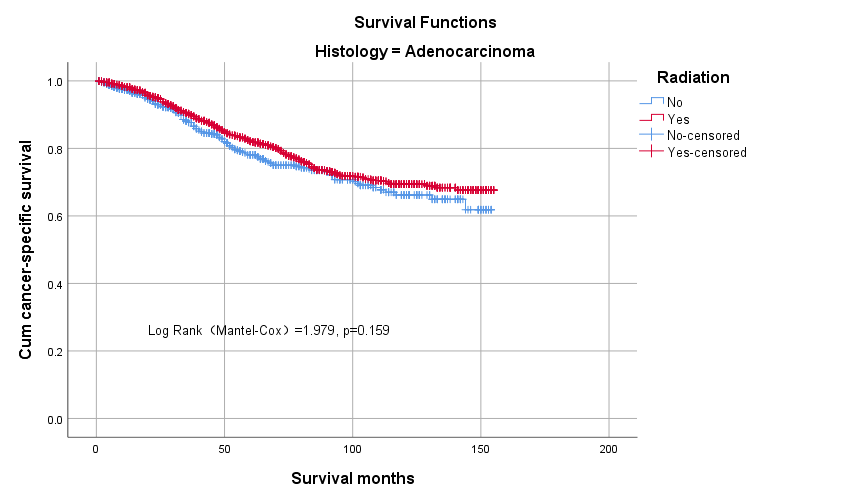

Supplement: Supplementary file 11 — Additional file 11. Figure 7A. Kaplan-Meier curves for cancer-specific survival (CSS) between radio and no-radio groups in adenocarcinoma. [file 12876_2023_2697_MOESM11_ESM.png]

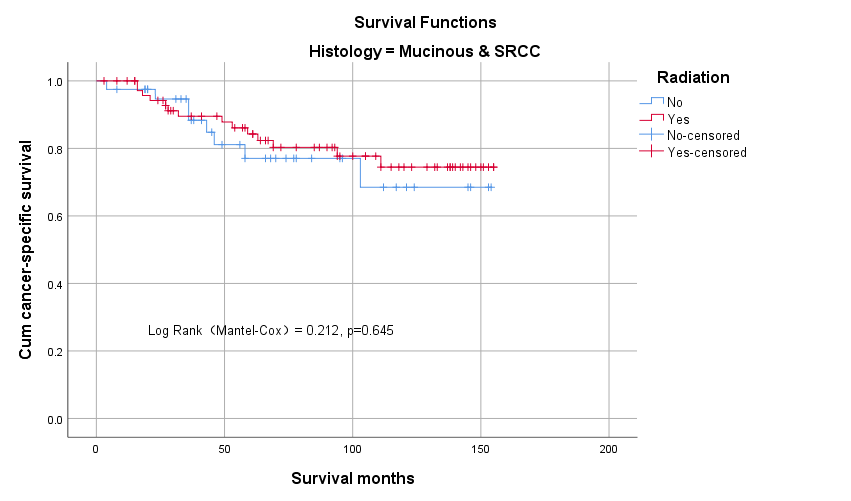

Supplement: Supplementary file 12 — Additional file 12. Figure 7B. Kaplan-Meier curves for cancer-specific survival (CSS) between radio and no-radio groups in mucinous & SRCC [file 12876_2023_2697_MOESM12_ESM.png]

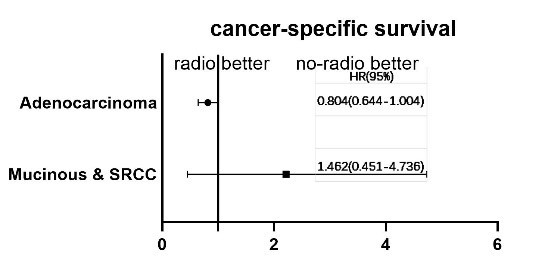

Supplement: Supplementary file 13 — Additional file 13. Figure 7C. Risk ratio of different histological type. [file 12876_2023_2697_MOESM13_ESM.jpg]
